# Notes of Surgical Cases: Paralysis of the Muscles of the Eye and Face from Concussion; Encephaloid Tumor; Lupus; Cases of Varicose Veins

**Published:** 1862-04

**Authors:** J. F. Miner


BUFFALO

### Medical and Surgical Journal

AND REPORTER.

VOL. I. APRIL, 1862. NO. 9.

### ORIGINAL COMMUNICATIONS.

ART. I.—Notes of Surgical Cases: *Paralysis of the Muscles of the eye and face from Concussion; Encephaloid Tumor; Lupus; Cases of Varicose Veins; by* J. F. Miner, M. D.

*Paralysis of the Muscles of the Eye and Face from Concussion.*—November 5, 1861: Visited L. Schriver, engineer, aged 26. The Tug upon which he was employed had been run into by a Propeller, and after the occurrence Mr. S. was found apparently dead, from injury he had received, in manner unknown. He had been visited by physicians while on the boat, who regarded him so nearly dead or so certain to die very immediately, that they left without action. He was removed to his residence where I found him insensible; pulse slow and scarcely perceptible. Surface cold; pupils dilated; head and face was covered with blood, which was flowing quite profusely from both ears and the nose; also spitting and vomiting blood, in great quantities. Respiration, slow and labored; deglutition difficult; eyelids distended with extravasated blood so immensely as to form the prominent appearance of injury; face and lips livid; occasionally slightly convulsed. Patient presenting the general symptoms of fracture at base of the skull, or the severest concussion, and a very guarded or unfavorable prognosis given. Stimulants and warmth were ordered, and the thought indulged that possibly reaction might yet take place.

*8th.*—Answers questions slowly; bleeding from the ears mostly subsided, though not wholly; sleeps heavily; pulse 40 per minute; respiration slow skin natural; comp lains ofno pain. Takes a little food and brandy in small quantities, every two hours.

12*th.*—Answers questions more readily; pulse 45 per minute; respiration more easy and natural; complains of pain and heat in the head; muttering delirium. Direct ice-bag to the head, which is very agreeable.

15*th.*—Pain in the head constant and severe; pulse 50 per minute and more full; sleepless and very restless. Half teaspoonful McMunn's Elixir every 4 hours, if necessary to obtain sleep; continue ice-bag to the head.

16*th.*—Has slept sufficiently; pulse 55 per minute and more natural; pain less severe; eyes can be seen through the small aperture we are able to force between the still greatly distended lids.

19*th.*—Eyelids more natural; convergent strabismus of both eyes, the external recti muscles being paralyzed; muscles of the face on the right side paralyzed; improving rapidly in general appearance.

*Jan.* 14*th,* 1862. Mr. S. presented himself at my office for examination, complaining of nothing except his eyes; the muscles of the face had regained their action, and he was in every respect healthy. Eyes greatly convergent; vision double; unable to do any work from imperfection and uncertainty of sight. Divided the tendon of the internal rectus of each eye, dissecting the conjunctiva and sub-conjunctival fascia freely from the inner third of the globe.

18*th.*—Vision greatly improved; is able to rotate the globe outward much better; result of operation perfect in the right eye, with slight convergence of the left eye.

It is impossible to know positively the exact character of the lesion present in this case, as indeed in all cases of concussion, since it has never yet been fully demonstrated what changes are produced in the cerebral structure. That the brain was itself the seat of injury, at least in part, seems highly probable, and yet it may not be certain that the nerves supplying the paralyzed muscles did not receive injury outside the cranial cavity. The general symptoms of fracture at the base of the skull were present in marked degree, and yet the result seems to throw much doubt upon the existence of such injury. The paralysis of the muscles of the left side of the face was well marked, though the control over them was never wholly lost. The strabismus was the only persistent paralysis, and possibly time might have proved sufficient remedy; yet there is but little ground for expectation in this, from the present condition of the patient.

The eyes seemed to take their normal position, after operation, without much contraction of the external muscles. Control over the motions of the globe are yet apparently imperfect.

*Encephaloid Tumor.*—*Nov.* 8, 1861. Miss —— aged 28, of healthy parents and general good health, offers for examination and removal a tumor situated upon the abdomen, at the right of the umbilicus. It has been noticed for ten years, but has increased rapidly for the past three months. It is movable, free from pain, nearly spherical, indistinctly fluctuating; exteriorly smooth, but upon pressure can discover irregularity as if lobulated. It is four inches in diameter and stands out very prominently. Assisted by Drs. Eastman, Nichell, Mason, and private students, the tumor was removed fully and perfectly. It was found to rest upon the abdominal muscles, and to be composed of a cyst, filled with bloody serum. The cyst itself was thick, and contained small cavities or cells; it was also noticed to be of a dark, livid color; this color extended through the superimposed tissue, and gave to the external appearance of the tumor a peculiar lividity, as if composed of many enlarged vessels. The wound healed kindly, though a tendency was noticed to bleed freely, especially on removal of the ligatures and sutures.

Three months after operation it is found again developing itself, a small, hard, livid tumor growing in the cicatrix. Pain is not much complained of; the girl looks pale, haggard and unhealthy.

At the present writing, six months after operation, the tumor is an open, bleeding, suppurating mass of encephaloid disease. It has several times bled very profusely, a pint in a short time. The pain is often severe, emaciation is rapidly increasing, and the case is soon to terminate.

We notice in this case the appearance, first of cystic tumor, with very few if any indications of malignant disease, though this form of disease is said to often present in early stages the cyst growth. That the operation should have been attended by success, in so far as the healing of the wound, and remaining so for some time, is concerned, is somewhat remarkable. The inquiry will be made, did encephaloid develope itself in the cicatrix? or was the cystic tumor malignant, and if unmolested, about to show its malignant character? There appears good ground for believing that the tumor removed would soon have shown the same appearances as the one now present, and that there was contained in it the germ of the present malady, from the earliest commencement of the disease.

Its location is somewhat unusual. Its rapid growth and present appearances show nothing of particular interest or of unusual importance.

The question is suggested: Do benign growths degenerate into malignant disease? It is claimed that such is the case, but it seems quite probable that the germ of malignant character is generally present from the commencement.

*Lupus.—Extirpation of the Globe of the Eye,*—Jan'y 1st, 1860. J. W——, aged 38 years; has suffered for the last twenty years with ulcers upon the face, neck, shoulders and breast. These surfaces now show the ravages of the disease; deep fissures and ridges are present, showing extensive and deep ulceration. The disease is now confined to the neck and face; the ulcers are partially covered by a brownish scab, and furshish an ichorous discharge. They heal sometimes at one margin, while at the other the ulceration is progressive. The surface which has been affected is red, and the vessels superficial, covered only by a very thin skin. The general appearance of the surface is like that left after a very severe burn. Has been healthy in other respects; has never had any other disease. The eye upon the left side has been the particular seat of the disease for the last three years, and constitutes the chief object of interest and attention at the present time. The conjunctiva is thickened and everted, so immensely distended as to form a tumor two and a half inches in diameter, covering completely the globe, and protruding out upon the face, a red, granular, vascular mass, which had been by some physicians regarded as fungus hematodes, or in other words, encephaloid disease of the eye. It furnishes an abundance of serum, sometimes tinged with blood, and causes a great increase in the glandular secretions of the eye, so that in all, the discharge is constant and exceedingly troublesome. The bones have also been involved in the ulcerative process; the orbital ridge of the frontal bone, and the frontal process of the malar bone had been very largely removed.

The pain, and activity of the disease, has always been paroxysmal; some times the pain was most intolerable during the periods of greatest activity. Under the influence of Iod. Potass and Fowler's Solution, the ulceration ceased, and in great degree the pain. The tumified conjunctiva being attended by so great deformity and inconvenience, and constituting in all so great a drain upon the system, it was resolved to dissect it out, and remove with it the lachrymal gland, which was done without difficulty. The pressure of the tumor and the effects of the disease has caused the upper eye-lid to become atrophied, and attached to the arch of the frontal bone, while the lower eye-lid had been pressed, and also drawn down by the cicatrix, so as to rest, and remain far down upon the malar bone. To remedy this deformity the lids were dissected up and drawn together. A transverse incision beneath the lower one being made, and a portion of the upper lip transplanted beneath the eye, to supply the deficiency of tissue. Thus arranged, the healing process accomplished, the eye was in very good condition as far as being covered, and washed by the lids. The cornea, however, was very imperfect, covered by deposite, vessels injected, and partially opake. After a few weeks, pain commenced again in the globe of the eye which nothing could abate. Every device being unavailing for this purpose, the globe of the eye was removed, a division, or amputation, being made about mid way the schlerotica. The pain at length abated, the stump and lids are in condition for artificial eye; the disease has never returned in the least, and seems to be permanently arrested.

This must be regarded as a very unusual case of disease. We have called it "Lupus," for the want of a better cognomen; and still there are objections to grouping all diseases of this character under one sir-name. It had been severe for twenty years, and under the care of different physicians, yet it abated very rapidly under the influence of eight grains Iod. Potass three times daily, and later, when Fowler's Solution was prescribed, all diseased action ceased. We are left ignorant of which remedial agent was most useful in this case, if indeed the improvement can be certainly attributed to either. The improvement commenced under the Iod. of Potass, and was completed while taking Fowler's Solution. The evidences are as strong that both agents were useful, as we generally obtain in the use of remedies. We have not seen or heard of Lupus affecting the bony tissue, unless we make lupus include the milder forms of malignant disease, in which case an arrest, or permanent cure, would be very remarkable. The great size of the tumor formed of the conjunctiva and of effusion behind that membrane constitutes an unusual feature. It is not unusual to see the conjunctiva distended and protruding forward, nearly or quite covering the cornea, as in the disease called chemosis; but to see such a tumor from thickening and effusion as we have attempted to describe, must be very infrequent. Removal of the globe of the eye for the relief of pain, caused perhaps by pressure from within its walls, is a practice which has quite frequently been adopted, and is only the carrying out of a principle often practiced upon not only in Ophthalmic Surgery, but also in many other cases, viz: relief of pressure. Perhaps this is not the true and full explanation in the case cited; possibly other causes might have increased the agony which had been long complained of, but certain it is, that this cause, if it was fully demonstrated to exist would be ample and satisfactory.

*Varicose Veins—Injection of per Sul. of Iron.* J. B——, Park Place. Under the case of Dr. Gould, by whose favor I am invited to operate for the obliteration of varicose veins. Patient is a carpenter by trade, healthy and robust. For the last five years has been greatly troubled by this disease in the left leg; pain severe while standing, and oftentimes large ulcers would compel him to keep the horizontal position for some time before they would heal.

*Sept.* 12, 1860.—We injected the internal suphenous vein in two places, separated by a distance of about three inches. Pressure was made above and below the joint of injection by the finger, so as to coagulate the contents of the vessel for the distance of about two inches. *Solution per sulphate iron two drops, water eight drops,* constituted the proportions of the solution, ten drops of which were used for each place. The blood was nsta ntaneously coagulated, and formed a perfect and complete "blockade" in the vessel.

This operation was unattended by pain of any importance or embarrassment of any sort. A warm fomentation was applied, and patient advised to keep the horizontal position.

About one week from the time of operation an incision was made down upon the hard coagulum, which formed a complete obstruction to the circulation, and it was removed. It now appeared a dry, hard substance, which could be broken and easily removed with the scalpel handle.

The distended vessel above and below had already disappeared, and nothing remained of the disease except the slight soreness at the points of injection. It is now eighteen months since the operation. Mr. B. has been mostly engaged in his usual business, and thus far it has not given him the slightest trouble.

Mrs. G——, aged 53 years. Admitted the second time to the ward of the Buffalo General Hospital for treatment of varicose ulcer.

*Jan.* 10, 1862, made injection into the internal saphenous vein of ten drops of dilute solution of per sulphate iron, making the injection only at one point. Patient placed in bed and fomentation applied.

11*th.*—Complains of pain and soreness. A general erythematous redness is observed on that side of the leg as high as the knee. The coagulum is large, and hard, and the tissues inflamed for a little distance above and below.

16*th.*—The redness, soreness and pain has abated. The ulcer is rapidly healing and the coagulum seems softening and undergoing the process of absorption.

25*th.*—Is around the ward; the varicose ulcer is healed; the vessel, so greatly distended, is no longer visible; some hardness yet remains at the point of injection.

*Feb'y* 5*th.*—Within the last ten days has been quite sick with erysipelas upon the face and ear, which cannot be in any way associated with the operation. Is to-day dismissed, having fully recovered, and commences labor as a house servant.

Michael ——, aged 26 years, healthy; has varicose ulcer upon the leg; has been troubled at times for several years.

*Feb'y* 20, 1862.—Injected the internal saphenous vein. In making the operation the point of the instrument was withdrawn too soon, allowing the escape of some small quantity of the per-sulphate iron, outside the vessel into the cellular tissue. Patient was placed in bed and poultice applied.

30*th.*—Very little pain or inconvenience from the operation. The vein is completely obliterated, both above for some distance, and below the point of injection. The old ulcer is healed. The cellular tissue and vein at the point where the operation was made have sloughed away, leaving a deep, granulating ulcer.

*March* 15.—Patient is well; and though the recovery has been delayed by the accidental escape of the solution outside the vessel, yet no very unpleasant effects followed, and the hardness and soreness usually complained of at the point of injection disappeared quite as rapidly as is usual in such operations.

These operations upon varicose veins have been related for the purpose of giving the results of some of the cases treated within the last few months. The course of treatment usually pursued for radical cure, has been, and perhaps still is, to apply ligature. We cannot yet properly compare the two operations for want of sufficient experience in the injection of per sulphate of iron. From what has thus far transpired in my own practice, or under my observation, I am more favorably regarding this mode of treatment, since I have never seen, or heard, of phlebitis arising in a single instance. The pain is less than usually attends the ligature, and the recovery is more rapid. We desire for the present to omit comment since our object was simply to give the facts as noted, and allow each one to draw his own inferences.

